# Expressing grief through metaphors: family caregivers’ experience of care and grief during the Covid-19 pandemic

**DOI:** 10.1080/17482631.2021.1996872

**Published:** 2021-10-29

**Authors:** Alexandra Guité-Verret, Melanie Vachon, Deborah Ummel, Emilie Lessard, Camille Francoeur-Carron

**Affiliations:** a Psychology Department, Université du Québec à Montréal, Montréal, Canada; b Center for Research and Intervention on Suicide, Ethical Issues and End-of-Life Practices, Montreal, Canada; c Réseau Québécois de Recherche en Soins Palliatifs et de fin de vie (RQSPAL), Quebec, Canada; d Psychoeducation Department, Université de Sherbrooke, Longueuil, Canada; e University of Montreal Hospital Research Center, Montreal, Canada

**Keywords:** Grief, bereavement, mourning, metaphor, Covid-19 pandemic, family caregivers

## Abstract

Purpose: The COVID-19 pandemic has disrupted thousands of individuals’ experience of caregiving and grief. This qualitative study aimed to gain in-dept understanding of family caregivers’ lived experiences of caregiving and bereavement in the context of the COVID-19 pandemic in Quebec, Canada. The study also aimed at providing new insight about caregiving and bereavement by analysing the metaphors family caregivers use to report their experiences. Methods: The design of this study was guided by an interpretative phenomenological approach. In-depth interviews were conducted with twenty bereaved family caregivers who had lost a loved one during the first waves of the pandemic. Results: Results indicate that bereaved family caregivers lived and understood their experience in terms of metaphoric cut-offs, obstructions and shockwaves. These three metaphors represented the grief process and the bereaved’s quest for social connection, narrative coherence and recognition. Conclusion: By identifying the meaning of the bereaved’s metaphors and the quest they reveal, our study underlines the singularity of pandemic grief and points to the value and meaning of caregiving with regard to the grieving process.

## Introduction

Upon submission of this article (September, 2021), nearly 1,6 million people had been infected with COVID-19 in Canada and more than twenty-seven thousand had died (Government of Canada, 2021a). As of today, thousands of individuals have become bereaved as the pandemic lingers on, disrupting many aspects of daily life, including funeral rites and social support (Doka, 1989; Wallace et al., 2020).

During Canada’s first wave of COVID-19, deaths among the over-70s accounted for more than 85% of all COVID-19 deaths (Government of Canada, 2021b). Moreover, elderly people who had some underlying health conditions were particularly vulnerable to the coronavirus disease, such as those who were living in nursing homes because these facilities were the first sites of viral outbreaks. About two-thirds of all COVID-19 deaths in Canada occurred in long-term care homes or in long-term care units (Canadian Institute for Health Information, 2021). Due to public health measures and restrictions required to protect the health system and reduce the spread of COVID-19 nationwide, visits to long-term care and personal care homes were no longer authorized. Under these extraordinary circumstances, thousands of people who had contracted the virus experienced suffering and isolation at the end of life (Vachon, 2021). It is also under such troubled circumstances that family caregivers began their painful journey, having been unable to accompany their loved ones until death and adequately commemorate them thereafter.

The death of a loved one represents one of the most difficult ordeals in life. For bereaved families and individuals, the world is turned upside down. Neimeyer’s (Neimeyer et al., 2010) work asserts that grieving is a process of meaning reconstruction in the wake of loss and that the loss is a disruption of the coherence of the individual’s self-narrative. This grieving process is complex and subjective, varying from one individual to another and from one moment to another for the same individual. However, we can assert that this process includes psychological, physical and social reactions, including a range of emotions such as sadness, despair, anger, guilt, loneliness, exhaustion and confusion (Zech, 2006). These common reactions to grief can be influenced by factors as diverse as the bereaved’s personality and life story, his/her relationship to the deceased, the circumstances of death, the support that is extended to the bereaved and even the cultural practices related to death and grief (Bacqué & Hanus, 2020; Zech, 2006).

Some clinical and theorical studies on grief has suggested that family caregivers are likely to experience a complicated grief in the times of COVID-19. For example, the cumulative effects of life stressors deriving from national health policies, the circumstances surrounding death and the lack of social recognition of death may make the grieving process extremely challenging for bereaved family caregivers (Vachon et al., 2020). Accordingly, they may experience suffering and guilt through the imbrication of multiples factors, such as the impossibility of being present at the bedside of the dying loved one, the obligation to remain distant, enable to touch him/her, the struggle to say goodbye, the absence at the time of death, the lack of preparation for death, the lack of social support fallowing death, the postponement of rituals and commemorations, the limited number of family members who can attend a funeral, and the social isolation of the bereaved in a period of confinement (Amy and Doka, 2021; Goveas & Shear, 2020; Kokou-Kpolou et al., 2020; Stroebe & Schut, 2021; Wallace et al., 2020). During this crisis, bereaved family caregivers could have great difficulty grieving and finding meaning to this loss, particularly if the accompaniment of the loved one at the end of life does not fit the idea of a “good death” (Wang et al., 2020; Wilson et al., 2016).

More broadly, recent research indicate that the coronavirus pandemic has caused major changes in accompaniment, end-of-life and bereavement experiences (Borghi & Menichetti, 2021; Hanna et al., 2021; Morris et al., 2020; Pattison, 2020; Vachon et al., 2020; Wang et al., 2020). Illness, mourning and death are now placed at the centre of Western societies in which those three issues are normally silenced or even denied (Bacqué & Hanus, 2020; Zimmerman, 2004). Therefore, it is possible the COVID-19 pandemic will lead to a unique awareness of the value and meaning of end-of-life support and funeral rites (Bermejo, 2020).

Finding meaning after the death of a loved one is an important part of the grieving process (Milman et al., 2019; Neimeyer et al., 2010). Grieving often means engaging in a search for meaning and coherence, as the experience of loss can force bereaved persons to question their entirely lives, for example, their identity, their beliefs, their life purpose, their future. To initiate and deepen this search for meaning during bereavement, the possibility for individuals to narrate and share their loss is determinant (Neimeyer et al., 2010). Indeed, recounting one’s experience of grief can facilitate the search for meaning. Grief is then transformed into a narrative that can be shared with others, integrated into one’s personal life story and eventually lead to a sense of cohesion (Vachon, 2021).

### Meaning and metaphors

Qualitative research suggest that metaphors can be a direct way of expressing, sharing and creating meaning in the process of grieving (Neimeyer et al., 2010; Young, 2008). The metaphor is an extremely efficient and sensible form of language capable of revealing the uniqueness of a lived experience. Metaphors can capture a complex reality in one single unit of meaning with a finesse all its own, so different from ordinary phrases. Paul Ricoeur’s (1975, 1986) hermeneutic phenomenological work asserted that lived experiences are only fully revealed through language, that is, through narratives and metaphors (Tuffour, 2017). According to Ricoeur (1986), the metaphor produces a gap in the ordinary use of words, destroys one order to create another (pp. 23, 39). The creation of a metaphor implies a new relationship to the world: the creation of metaphors is the creation of new meanings and therefore the emergence of a new way of questioning and living our world (Ricoeur, 1975, p. 369). Thus, speaking from an hermeneutic phenomenological view, the core function of a metaphor is to express and share a lived experience in a novel, “vivid” way (Ricoeur, 1975). Therefore, within a shared personal narrative, metaphors represent a fundamental path of experience and a search for meaning.

This conceptualization provides a better understanding of the methodological potential of metaphors for qualitative research, or for interpretative phenomenological research in particular. Conducting a study with a focus on metaphorical language may open up creative avenues and allows for new insights and understandings to emerge (Shinebourne & Smith, 2010).

Likewise, by means of this study, we believe metaphors can disclose new aspects of the experience of family caregivers as they cope with grief. Because metaphors allow for a detailed and nuanced knowledge of a person’s experience, we believe they represent a relevant avenue to derive a better understanding of the lived experience of bereavement during the COVID-19 pandemic. Recently, Stanley et al. (2021) conducted a metaphor analysis of the COVID-19 pandemic and provided insight into peoples’ collective trauma. Their qualitative study suggests that individuals can articulate deep implicit emotions about their pandemic experiences by sharing and reflecting on metaphors related to the traumatic events. However, to the best of our knowledge, no studies have yet explored the metaphors of bereavement with regard to the COVID-19 pandemic.

### Aims

This study aimed for an in-depth understanding of family caregivers’ lived experience of caregiving and bereavement during this pandemic, by analysing the metaphors they created when recounting their grief journey.

This study is embedded within a wider qualitative project on individuals’ experience of grief in the times of COVID-19 (Vachon et al., 2020), and led by researchers from Montreal, Canada. This wider project is not based in clinical practice, even though it allows research participants to narrate their experience in a research context while receiving human support from a committed listener with grief experience.

## Methods

### Study design

On a methodological and theorical level, this study is anchored in Interpretative phenomenological analysis (IPA) (Antoine & Smith, 2017; Smith et al., 2009; Tuffour, 2017). Interpretative phenomenological analysis is an approach, a theory and a research method. It aims to explore, describe, interpret and situate the participants’ personal experiences (Tuffour, 2017). IPA aims precisely to capture how people make sense of their lived experiences, with a focus on personal meaning in a particular context (Smith et al., 2009). This refers to a “double hermeneutic” in which the researcher interprets the way an individual interprets his/her own experience (Smith et al., 2009; Tuffour, 2017). The participant’s interpretation is first-order, while the researcher’s interpretation is second-order (Smith et al., 2009). To access the deeper meaning of an experience, researchers have to let themselves enter the participant’s life world and, more abstractly, be open to otherness (Depraz, 2012). From this perspective, the research starts “from connection instead of detachment” (van Wijngaarden et al., 2017, p. 1740).

As IPA is strongly influenced by the hermeneutic version of phenomenology, it rejects the idea of producing a pure description of a phenomenon. IPA stipulates that researchers always interpret of the phenomenon they study, and that a reflexive practice is always required to acknowledge this interpretation (Smith et al., 2009). Researchers must be transparent and sincere about their methodological and theoretical assumptions (Tracy, 2010), as much as they need to acknowledge their subjectivity by being reflexively aware of their own ontological and epistemological stances (Willig, 2012). In practice, we saw our own process of perception and understanding by keeping a reflexive journal and by discussing with peers.

This study aims to gain insight into the participants’ personal sense-making from the metaphors they create. According to Ricoeur (1986), metaphors produce a deviation in the usual use of words, destroy one order to create another, which leads to a re-description of reality and an unprecedented relationship to the life world (pp. 21–28). The creation of metaphors is the creation of new meanings and therefore the emergence of a new way of questioning and living our world (Ricoeur, 1975, p. 369). Ricoeur (1996) claims that every individual has an innate capacity to engage in personal sense-making and to create metaphors (p. 24). This human capacity allows each of us to deepen our understanding about our lived experiences and the way our experiences are linked with a common meaning.

### Participants

The study sample included 20 participants, including 17 women and 3 men. If this sample is mainly composed of women, it is a representative one since the vast majority of family caregivers are women (Washington et al., 2015). The participants were contacted on the project’s Facebook page and from the authors contact networks. The criteria of inclusion were (1) being no younger than 18 years old and (2) having experienced the death of a loved one during the COVID-19 pandemic. The recruited participants had lost a loved one during the first wave of the pandemic (spring 2020), except for one participant who faced the loss during the second wave (fall 2020). We can specify that the participants were not already involved in a clinical practice for bereaved affected by the loss of a loved one through COVID 19. Table I provides more characteristics of the sample.

**Table I.** Characteristics of the participants

Characteristics	*N* (%)	*M* (SD)
**Sex/gender**		
Women	17 (85)	
Men	3 (15)	
**Age** (years; range: 21–78)		54.2 (14,7)
**Time after the loss** (days; range 12–237)		95.4 (74,6)
**Relationship with the deceased**		
Mother	7 (35)	
Father	6 (30)	
Spouse	5 (25)	
Grand-parent	2 (10)	
**Civil status**		
Single	4 (20)	
Married/cohabiting	10 (50)	
Separated/divorced/widowed	3 (15)	
**Death location**		
Hospital	14 (70)	
Residence for elderly	6 (30)	
**Dementia comorbidity** No Yes **Commemoration**	14 (70) 6 (30)	
Nothing	2 (10)	
Virtual	6 (30)	
In person	12 (60)	
**Possible visit at bedside**		
No	13 (65)	
Yes	7 (35)	
**Interview length** (minutes; range:11–91)		60.6 (28,9)
**Number of interview(s)**		
1	10 (50)	
2	10 (50)	

### Ethical considerations

This study received the approval of the ethics committees (nº 2020–2590) of Université du Québec à Montréal (Montreal, QC, Canada) and Université de Sherbrooke (Longueuil, QC, Canada) where it took place. All participants were given a full explanation of the study’s purpose, the voluntary nature of their participation and their freedom to stop the interview or to withdraw at any time. All participants were also assured of confidentiality and integrity. Yet some participants (*n = 12*) wanted their real names to appear in this article in order to go public and have their grief acknowledged. Written or verbally informed consent was obtained of each participant.

### Procedure and settings

Data collection took place from May to November 2020. In-depth, individual interviews were conducted by trained researchers, including second and third authors. Each of the participants were interviewed once or twice, and each interview lasted 60–90 min. In line with the IPA approach (Smith et al., 2009), an open question was used to invite participants to describe in dept how they lived both the experience of caregiving and the grieving process in the context of the COVID-19 pandemic. We invited the participants to develop their personal stories from the following question: “Can you tell me what happened to you from the moment you suspected the contamination of your loved one, until today?”. The participants were encouraged by probes such as, what was it like? How did/do you feel about this? How do you understand this? What was difficult about this? What happened then? Each participant was also questioned about key events like the coronavirus diagnosis, the circumstances of the death and the mourning rites, if those events had not been spontaneously described by the participant. All interviews were audiotaped and transcribed verbatim. Moreover, as an interpretative phenomenological approach was used to guide both the data collection and the data analysis, reflexive notes were taken after each interview and discussed between interviewers and authors.

Reflexive notes were taken after each interview and discussed between interviewers and authors. Reflexive notes were also taken by the first author during data analysis and discussed with the research team to ensure dialogue around participants’ experience.

### Data analysis

Interpretative phenomenological analysis (IPA) (Smith, 2007; Smith et al., 2009; Tuffour, 2017) was used to analyse the interview transcripts. IPA is a flexible and iterative process of analysis rather than a rigid and linear method (Antoine & Smith, 2017; Smith et al., 2009). With this in mind, the IPA guideline involves seven steps: (1) reading and rereading the first case, (2) initial noting, (3) developing emergent themes, (4) searching for connections across emergent themes, (5) moving to the next case, (6) repeating step 1 to 4 for each case, (7) looking for patterns across cases.

In conformity with the IPA concept of “double hermeneutic”, we interpreted how the phenomenon of grieving was interpreted by each participant, based on the metaphors each one spontaneously created during the interview. We also assumed that a first hermeneutic consisted in engaging with the metaphors created by the participants, while the second hermeneutic consisted in interpreting the meaning of those metaphors with regard to the grieving process.

Furthermore, as IPA is an idiographic approach, we considered each case individually and read each of them repeatedly before proceeding to the analysis of the convergence and the divergence between cases. The process of interpretation was understood as a “hermeneutic circle” and was concerned with the dynamic relationship between the part and the whole, between the text and the textual interpretation (Smith, 2007). At another level, the data analysis was concerned with the dynamic between researcher and participant, between the researcher’s preconceptions and experience and the encounter with a participant (Larkin et al., 2006; Smith, 2007). The IPA analysis provided and relied on a range of different levels and different ways of thinking about the data, giving access to multiple layers of meaning (Smith et al., 2009). Nevertheless, as the hermeneutic circle speaks to a non-linear type of analysis, our understanding remains open to new insights, and it was up to the researcher to decide when the interpretation was satisfactory (Smith, 2007).

### Rigour

To ensure the rigour of this study, we followed Tracy’s (2010) eight criteria of quality in qualitative research, as listed and exemplified in Table II.

**Table II.** Tracy’s eight criteria of quality in qualitative research

Criteria for quality	Various means and practices through which criteria were achieved
Worthy topic	Relevance with regard to societal events and priorities In line with recent literature on bereavement Opens up new avenues of design (metaphorical analysis)
Rich rigour	In-dept interviews; high level of transcription details Abundant and complex data Appropriate and complex theorical constructs Transparency about data collection and data analysis Discussion with peers
Sincerity	Self-reflexivity about the data analysis and the researchers’ pre-conceptions (reflexive diary and discussion with peers) Transparency about methodological and theoretical assumptions Recognition of the study limitations
Credibility	Thick description (situated meanings; abundant quotes; illustration of the data’s complexity) Immersion in the data to ascertain tacit knowledge Crystallization (embedment of the study within a wider project; multiple researchers and multiple data sources)
Resonance	Evocative stories and metaphorical representations Transferable findings
Significant contribution	Practically significant research (sheds light on the experience of grief in the context of the COVID-19 pandemic) Heuristic and methodological significance (underlines the potential of the metaphors on a methodological level; provides extending knowledge on the metaphors of bereavement) Findings can lead to improve clinical practices
Ethics	Procedural ethics (boards of ethics’ approval) Situational ethics (reflexive ethical practices; recognition of the participants’ specific context) Relational ethics (commitment and availability of researchers; recognition of interdependence between researchers and participants)
Meaningful coherence	Questions, paradigm, method and analysis in line with IPA Interconnections between stated goals, literature, data and interpretations

## Results

The metaphors that participants used in their personal stories shed light on the meaning of their experience. Our interpretative phenomenological analysis allowed the identification of three metaphors that configured the bereaved’s narratives and carried the meaning of caregiving and grieving in the context of COVID-19 pandemic: (1) being cut off from others, (2) living and facing obstructions and (3) feeling shockwaves. According to the meaning of these metaphors, the participants appeared to be engaged in a search for (1) social connection, (2) narrative coherence and (3) recognition. The following section details the description of these three metaphors and their meaning with regard to the grieving process.

### Being cut off from others

All participants seemed to live and understand their experience of caregiving and bereavement as a metaphorical “cut off” form others, whether it be their loved one now deceased, their relatives and friends, the clinicians or the funeral homes that carried out the body’s cremation. The metaphor of cut-off found in the bereaved’ stories postulated a state of forced social isolation and reflected both a lack of communication and a lack of physical, emotional and spiritual connection with others.

Most participants began their story by sharing experiences in which they felt powerlessness because they had not heard from the loved one from the time he/she tested positive for COVID-19. Most participants recalled that the diagnosis triggered in them an overwhelming concern about the loved one. This concern worsened over time because of the visitor restrictions in hospital units or care homes and because of the unavailability of the clinicians who were the only ones in contact with the loved one. It seemed that the participants’ suffering stemmed from a double impression of powerlessness and exclusion regarding the loved one who they imagined as being in great pain and danger. As such, being cut off from the loved one meant the inability to take care of him/her, to reassure him/her and to be in “the heart of the action” in order to ensure the quality of his/her medical care:

It was the first, how could I say, the biggest, uh, cut-off ! You know … not being with my father. (Caroline)

And that was the fatal date for the closing of hospitals and care homes. So, as a caregiver, it was … We are no longer in the picture. That’s where it starts. For us the horror movie begins there. (Marie-Hélène)

It appeared to us that being cut off from the loved one also meant the impossibility of accompanying the dying. The cut-off experienced by most participants seemed to have generated feelings of powerlessness, anger, regret, guilt, hopelessness. Several participants learned about the death of the relative over the phone, often hours, even days after death had occurred. These painful circumstances marked the beginning of the grieving process. This also resonated with the first impossibly of visiting the loved one and being truly present with him/her throughout the final weeks and days of life. As a result, many participants felt they had abandoned the loved one at an essential “passage” of life, that is, death. In addition, participants often recalled the profound consequences to the quality of end-of-life due to social cut-off. The bereaved were often “haunted” by the idea that their relative may have died in a state of loneliness, sadness, or incomprehension, especially if he/she had dementia. Tamara stated the following:

My father died all alone, […] he was even abandoned for three months, isolated from his family, […] it’s horrible! You know, I mean, it’s, this, this, this, this, it breaks my heart, knowing that my dad could have felt like this. […] He knew what was happening […] but that does not replace the emotional emptiness he must have felt. (Tamara)

A metaphorical cut-off was also experienced by participants who could visit the loved one at the bedside shortly before death. This last visit represented a cut-off because, as expressed by many participants, accompanying a person suffering or dying goes beyond just being physically present. Wearing protective equipment, rushing goodbyes, doubting that the other understood us because he/she seemed unconscious, not being able to touch or give a warm embrace … The bereaved’ stories showed an extensive suffering and reported situations in which participants were not able “to really be there”. Sophie’s story suggests that the experience of social cut-off led to a feeling of inner “destruction”, close to an existential “collapse”:

My grandmother was lying on her deathbed … And I couldn’t give her a hug. […] I put my arms around her, you know on her, her arms, because I wore gloves and a gown. But she tried to give me a hug and I looked at her and I said “I can’t” … And you could see that it was destroying her, and that was also destroying me … (Sophie)

After the death of the loved ones, the social cut-off between caregivers and those around them illustrated both the difficulty of sharing suffering and the impossibility of having adequate support. The bereaved mentioned that conversations via phone or video chat apps, and virtual or limited funerals represented significant but incomplete relationships, because they had to maintain a distance from others. This is what Louise seemed to express when using the metaphorical image of a “filter”, symbolizing the thin barrier she felt between her and those around her at her dad’s funeral:

Of course, these are exceptional circumstances, but … well, sure … it’s, it’s more distant … […] Despite the rituals, eh, we wore masks … […] That makes it, a, a little, uh, a uh … not a limit, but … a little filter … (Louise)

Our analysis indicates that participants felt a strong need for human connections, whether before, during or after the death of the loved one. Despite this need, the presence with the loved one before his/her death was limited, just as the sharing of the experience of loss was limited after his/her death. As such, our analysis brought to light how the participants have sought to reconnect with others each time social connections were cut off. During the interviews, this search for connection appeared to be still in progress.

### Facing obstructions: blocked cares and blocked grief

Most participants seemed to considerably suffer from two major “obstructions” they experienced. First, their cares to the loved one had been “blocked”, that is abruptly stopped after he/she had contracted the virus and had been isolated in his/her room. Later on, after the death of the loved one, the participants felt they were living a “blocked” or “suspended” grief.

In the first instance, the bereaved family caregivers used the metaphor of obstruction to express a feeling of having lost track of events, or having lost contact with “the chain of events” when hospitals and other facilities were limiting or prohibiting visits by caregivers to their loved ones. In fact, before the COVID-19 pandemic, a vast majority of the participants were closely participating in the daily cares of their loved ones. By contrast, the ban on visiting him/her put an obstacle in their relationship and more broadly in their common caregiving story. The proven care abruptly stopped, blocked from then on. Some participants interpreted this by saying that “a part of the story” was missing or that the “last piece of the puzzle” was missing. In that sense, the metaphoric domain of obstruction shed light on the bereaved’s attempt to reconstruct the last moments of their loved one’s life in order to better understand what happened to him/her when he/she was alone facing the disease during hospitalization or in a care home. This narrative reconstruction also seemed crucial to gain an inner representation of the circumstances under which he/she had died or had been cremated. For example, Bertin recalled the moment when he became excluded from his mother’s life and thus incapable of knowing what she had lived through:

And well, we had a lot of trouble communicating. I don’t know when [my sister] communicated with mom … We kind of lost track … I didn’t manage to talk to her again until the day before her death, it was uh … was hell … (Bertin)

Our analysis also suggests that facing death in the context of the COVID-19 pandemic had extensive negative impacts on caregiving experiences and end-of-life plans. Most participants had done end-of-life planning with the loved one and had thought they’d be present, sitting at the bedside, perhaps holding the hand of the person. So, if they expected to ease suffering, improve quality of life and accompany the person towards death, the COVID-19 pandemic had completely disrupted care plans and had thus generated a need for some coherence between the planned accompaniment and the unexpected end-of-life. However, this need was difficult to fulfill, as Tamara stated about her dad’s death:

It breaks my heart because we’ve been there from the start, you know, and I would have been there, you know, I would have been there with him every day … […] I feel like there’s something that had been unfinished […]. I don’t know, it’s like, I am doing a puzzle and I am missing the … There is a feeling of, of no-ending that haunts me … (Tamara)

Rachelle’s narrative also suggested that the disruption in the care provided to the loved one especially affected the integration and the meaning of his/her death:

You know, we had our end-of-life plan with my dad […] and the coronavirus just burnt it all down. […] There’s this nuance … Yeah, like, we’ve had a double end of life. (Rachelle)

An analysis of participants’ accounts clarified how some bereaved caregivers had to integrate a “double” death: the expected death in conformity with advanced care plans, and the unexpected death conceived from the upheaval of family caregiving expectations. The metaphoric expression of “burnt care plans” revealed the violent nature of the lived experience of loss during COVID-19.

In the second instance, a vast majority of the participants used the metaphor of obstruction to describe their experience of “blocked” or “suspended” grief. They emotionally recounted that they did not witness the death and/or did not see the deceased’s body. Many actually felt that the death of the loved one was “unreal”, not truly realized, and therefore not really integrated. Again, a part of the story is missing, that of the death itself, and there remained a need for continuing the story in order to reconstruct it into a complete meaningful narration. The blocked grieving, as part of a lack of knowledge and understanding of the loved one’s death, draws out the meaning of the bereaved’s need “to resolve” grief and “stop waiting” for the funeral. This was needed to “move on” and “come full circle”, that is, to move forward in the grieving process and redefine the relationship with the loved one now deceased:

I think my frustration really comes from the impossibility of doing the work of grief as one should do it, with, with, well, with the ritual, which is, which is very healing and which allows us to … turn the page. (Bertin)

I, I don’t know, it’s like a blockage. It’s like there’s something that is caught inside of me and that is, that is, that is not going away. I, I, I dream of, of the day when I will be able to stand in front of his funeral urn and really … talk to him. To tell him, tell him what I feel, to tell him how important he was for me. And finally to say goodbye to him, because I haven’t been able to say goodbye … (Annie)

Most family caregivers also reported feeling powerless when facing the fact that they had lost the momentum of funeral rituals, the public commemoration of the loved one having been postponed. They reflected on the importance of being able to commemorate the loved one “in continuity with” his/her death. Indeed, planning or creating new rituals seemed to be one key strategy for addressing this blocked grief and giving meaning to death and grief. This may have represented a promise of coherence to the participants with regard to the death and also to the life to come without the presence of the other.

It appeared to us that, through the metaphor of obstruction, most participants sought and were still seeking to fill in the gaps in the loved one’s story in order to establish a better continuity between (1) accompaniment and death, and (2) death and grief. Their quest for narrative coherence appeared significant because the lack of continuity led to a grief “put on hold”.

### Feeling shockwaves

Our analysis also allowed the identification of the metaphor of shockwaves created by the bereaved to express how pandemic grief was especially devastating and unlike any other grief. The shockwaves metaphor revealed an additive process. Indeed, the participants lived and understood the experience of grief as an accumulation of shocks. This meant that feeling shockwaves was a unique experience, unlike the single shock commonly used to qualify the grieving process in normal times. Many participants shared having experienced “shock wave after shock wave” from the time their loved one’s life became threatened by the coronavirus. In addition, many recounted the multiple shocks they felt and the personal “collapse” that these shocks could cause each time. These shocks, followed by these collapses, were all metaphoric stages that participants had to go through, from caregiving to grieving. From this perspective, it seems that the pandemic grief was lived as an increased suffering:

I first had a positive idea. I said to myself: well, well, ok, she’s going to be fine, because she is tough and then … And then, I felt the first shock. And then there was the second shock because of the protocol, and then … Then, you say to yourself: ok, that’s it, it’s over, you go through it and … And then the horrible phone call … […] when my sister called me, well it’s … you collapse again. There were actually three collapses […]. It hurts each time as much as before. It was very … There, there was a kind of gradation in the violence. (Bertin)

It was a triple shock … […] In fact, it is because he was not so bad. Then he had COVID. We did not see him, then he died suddenly, it is like … We didn’t even have time to realize he wasn’t feeling good. It’s, it’s … a dual shock … I don’t know, I, I, I say triple because it’s, it’s is a lot … (Perla)

For the bereaved, the shockwaves metaphor has the power to express the drastic, violent, unexpected nature of the chain of events surrounding the death of the loved one.

Furthermore, a vast majority of the participants situated their intimate suffering within the social context of the pandemic in such a way as to distinguish themselves from the general population, which was also experiencing several losses. In the same vein, they clearly distinguished their current grief from other griefs they had lived in the past:

Grieving is one thing, social confinement is, is, is something else that gets on top of it all, uh … that adds another difficulty. (Suzanne)

You know, basically what we’re going through is not just grieving, it’s grieving in a pandemic. (Isabelle)

Most participants spoke of their experience metaphorically as “above” the experience they associated with other people. The social context of the COVID-19 pandemic, by its gravity, was often experienced by the participants as a pervasive context that kept them away from their grief, alienated them or even delegitimized their personal suffering. This is what Annie seemed to express by using the metaphor of the “burial” to better describe how she experienced her grandmother’s death:

Uh, my grandmother, well, was an elderly person so uh, an elderly person who dies … A … We, we expect it … And a person who dies in the pandemic, we also expect it a little, so yeah that’s it, she’s been kind of buried by a lot of things … Uh, and maybe our family, actually, me, I, I, I try to, to remove things, day after day, to, to make room for her … (Annie)

By describing their experience of grief and by interpreting it in relation to a “normal” grief, the participants sought to better understand their painful experience. The image of shockwaves took on a much more painful meaning than the image of a single shock and thus expressed more accurately the lived experience. In that sense, our analysis highlights how much it matters to the bereaved to describe, understand and share with others the meaning of an excess of suffering within the grieving process. The metaphor of shockwaves revealed this quest for recognition of the unique suffering and grieving they experienced during COVID-19.

## Discussion

This study aimed for a better understanding of the experience of grief among family caregivers who lost a loved one in the context of the COVID-19 pandemic. Our analysis suggests that participants lived and understood their experience of caregiving and grieving in terms of metaphoric cut-off, obstruction and shockwaves. These three metaphoric domains shed light on the meaning of the lived grieving process. The metaphors also revealed the participants’ desire to find meaning through a triple search for social connection, narrative coherence and recognition.

Our findings highlight family caregivers’ experience of being cut off, as they lived and suffered from social disconnection. This study provides in-depth descriptions of a wide range of experiences related to this disconnection, namely the lack of interaction and communication (1) between family caregivers and their loved ones, (2) between family caregivers and clinicians (both before and after death) and (3) between family caregivers and their relatives and friends (after death). In line with previous studies conducted on pandemic grief (Downar et al., 2020; Mayland et al., 2020; Stroebe & Schut, 2021; Vachon et al., 2021), we found that living social cut-offs impacts the whole process of grieving, because a specific forms of incomprehension, powerlessness, frustration and guilt stemmed from both the disrupted caregiving and the isolated end-of-life of the loved one.

Our study is significant in that it provides a description of the family caregivers’ experiences of social disconnection. From a hermeneutic phenological view, the metaphor of cut-off reveals an altered mode of being related to the world and to the others. Whether it concerned the need to be present at the bedside of a loved one or to share rituals after his/her death, the participants in this study sought for an “embodied” connection, for an authentic exchange based on proximity and reciprocity. In line with this view, Merleau-Ponty’s (2013) phenomenological notion of “intercorporeality” is relevant, as it points to the crucial role of the embodied links that support and underlie our relationships with others (Harrison et al., 2019). Bereaved family caregivers can deeply feel the lack of connection with others when it is impossible to coexist with them or to touch them in order to either express what should be said or do what should be done. Being in an intercorporeal relationship with the loved one and “being really present” seems to be part of a search for meaningful communication with the other as death approaches (Burrell & Selman, 2020; Holm et al., 2019; Otani et al., 2017; Pattison, 2020). Interactions that are shaped by a particular attention to the “the lived body” (Merleau-Ponty, 2013), through which we enter the world and profoundly meet others, also establish, for caregivers, a particular ethics of care in the face of life and death (Vachon, 2019). Moreover, the lack of human embodied connection can be experienced by family caregivers when they face the unavailability of clinicians. During the first wave of COVID-19, it was often difficult to connect with clinicians and thus to feel their compassion. Our results call for support initiatives and interventions aimed at promoting social connections among clinicians, patients and their families, because as we know the social and emotional support of relatives and clinicians is crucial to bereaved persons (Bandini, 2020; Chan & Chan, 2011; Rodger et al., 2007; Wilson et al., 2016).

In this study, family caregivers recounted their grief starting from their caregiving story with the loved one. This story often relied on feelings of closeness, commitment and presence, which had nothing to do with the experience of being cut off during the final weeks of his/her life. Scientific literature indicates that commitment to end-of-life care decisions and preparation for death are beneficial for family caregivers both during the hospitalization and afterwards in bereavement (Bandini, 2020; Breen et al., 2018; Mitima-Verloop et al., 2021). During the first wave of the COVID-19 pandemic, those significant actions were limited, which may have left family caregivers with a strong impression of living a blocked, disrupted relationship with the loved one. This brutal unanticipated loss of the loved one can lead to a negative view of death and may possibly alter the integration and the meaning of his/her death (Wilson et al., 2016). In our study, the bereaved’s view of the death emphasized pain and loneliness, and thus did not fit in with their idea of a “good death”. Previous studies underline how important it is for family caregivers to provide what they feel is a “good enough” accompaniment so they can transcend death and give meaning to life without the departed (Totman et al., 2015; Vachon, 2020). As such, the accompaniment of ill or dying persons during COVID-19 pandemic can lead to a quest for narrative coherence and a search for meaning in such troubled times.

Moreover, the bereaved family caregivers in our study used the image of “blocked” grief to express their concerns about rites and funeral practices. They described how being excluded from the loved one’s death caused feelings of powerlessness, uncertainty and confusion. Seeing or taking care of his/her body was prohibited, as well as organizing a public commemoration to honour his/her life. In normal times, the final separation permitted by sight, body care and funeral rites is crucial and meaningful because it marks the transformation of the relationship with the deceased and comes to fix the place of the dead and the living (Romano, 2015). In contrast, our results suggest that the absence of the body can be a distressing part of family experiences of grief when it prevents them from reintegrating the loved one as a dead person with whom the relationship become an abstract, symbolic one (Bacqué, 2020). Our results also suggest that postponed funerals may lead to “blocked” grief because of the impossibly of pursuing the social act of mourning. Every culture has its own customs and rituals for mourning loved ones and to metabolize and make sense of discontinuities and obstructions relating to the deaths (Des Aulniers, 2007). In this study, the undermining of the cultural customs and rituals was not only painful but was a real “obstacle” to the integration of death. It appears to us that incomplete accompaniment and funeral rites may accentuate the ambiguity the bereaved persons often experience between the absence and the presence of the deceased (Breen et al., 2018; Fuchs, 2018). The fact that one participant in our study referred to her mother as a “missing person” is a good illustration of this issue and a concrete example of blocked grief.

Our findings also suggest that it is important for bereaved family caregivers to express the particularities of their grief and suffering. If the shock metaphor is often cited in scientific literature as a common experience at the beginning of the grieving process (Bacqué & Hanus, 2020; Zech, 2006), the shockwaves metaphor expresses a unique experience and may describe what distinguishes pandemic grief from “normal” grief. Our study indicates that the suffering caused by multiple shocks can extend to every moment surrounding the death of the loved one. In the narratives we study, the bereaved described and understood the phenomenon of pandemic grief as an excess of suffering to cope with and as an excess of obstacles to face. The experience of shockwaves may usher in a quest for recognition of their grief in all its singularities and multiple layers of meaning. The lived experience of bereaved family caregivers is close to what Doka (1989) names a “disenfranchised grief”, that is a death that is not openly acknowledged, socially validated, or publicly mourned. In this regard, our results call for the unveiling of an “epidemic of grief” within the COVID-19 epidemic (Pearce et al., 2021; Petry et al., 2020). With that in mind, we found significant that some of the participants wanted to use their own names in the publication of this article. The participation in this research thus seems to have alleviate the suffering of anonymity and the non-recognition of bereavement.

Finally, these findings are in line with other studies that underline the risk for the bereaved when the meaning and value of funeral rites are underestimated, and when the social nature of grief is trivialized and death striped of its singularity (Kokou-Kpolou et al., 2020; Vachon et al., 2020; Wallace et al., 2020).

## Methodological considerations

To our knowledge, this study is the first to explore the metaphors used by bereaved family caregivers who have lost a loved one during COVID-19. Though this study adds new insight to the existing literature on pandemic grief, it can be nuanced by its limitations. The sample was limited to people who had experienced loss during the first wave of the COVID-19 pandemic. In this specific context, public health measures were extremely restrictive and the elderly living in care homes were the principal victims of the coronavirus. It is possible that a different context, for example, a less restrictive context in terms of patient accompaniment, social distancing or funeral practices, comes to modulate the experience of the bereaved and their intimate search within the grieving process. The findings transferability is thus limited by the sample’s homogeneity and the particular context in which understanding emerged. However, the rich details we have obtained from IPA can provide information to clinicians on how to better communicate and support family caregivers before and after the loss of their loved ones.

Nevertheless, the understanding allowed by an interpretative phenomenological approach has to be judged and appreciated with regard to a constructivist-interpretative paradigm. Thus, the quality of this study is based on the meaning that emerged from the descriptions of the participants’ stories, as well as on the openness and reflexivity of the authors (Tracy, 2010; van Wijngaarden et al., 2017). We also argue that the density and the complexity of the stories collected, as well as the credibility of their presentation in this article, contribute to the overall rigour of this research (Tracy, 2010).

## Conclusion

The COVID-19 pandemic has brought about major changes in the accompaniment of the end of life, which in turn have changed the way of living and mourning a loved one. This study’s interpretative phenomenological analysis reveals important metaphorical dimensions of the experiences of accompaniment and grief in this context: the search for social connections, arising from the multiple cut-offs experienced with others; the search for narrative coherence, arising from a disrupted accompaniment and a blocked grief; the search for recognition, arising from a need of expressing, sharing and making sense of the shockwaves felt throughout the pandemic. This study also reveals how the feeling of powerlessness and the difficulty to give meaning to death are at the very heart of these experiences. These findings can inspire future clinical interventions that would promote patients and families’ connection and encourage the bereaved to tell the story of the death of their loved one in order to make it more consistent and meaningful. On an institutional and cultural level, this study calls for the recognition of pandemic grief through a recognition of the meaning and value of both caregiving at the end of life and funeral rites after death.
